# Total Laboratory Automation for Rapid Detection and Identification of Microorganisms and Their Antimicrobial Resistance Profiles

**DOI:** 10.3389/fcimb.2022.807668

**Published:** 2022-02-03

**Authors:** Abdessalam Cherkaoui, Jacques Schrenzel

**Affiliations:** ^1^ Bacteriology Laboratory, Division of Laboratory Medicine, Department of Diagnostics, Geneva University Hospitals, Geneva, Switzerland; ^2^ Genomic Research Laboratory, Division of Infectious Diseases, Department of Medicine, Faculty of Medicine, Geneva University Hospitals, Geneva, Switzerland

**Keywords:** total laboratory automation, antimicrobial susceptibility testing, Copan, WASPLab^®^ platform, WASPLab artificial intelligence, Colibri, Radian

## Abstract

At a time when diagnostic bacteriological testing procedures have become more complex and their associated costs are steadily increasing, the expected benefits of Total laboratory automation (TLA) cannot just be a simple transposition of the traditional manual procedures used to process clinical specimens. In contrast, automation should drive a fundamental change in the laboratory workflow and prompt users to reconsider all the approaches currently used in the diagnostic work-up including the accurate identification of pathogens and the antimicrobial susceptibility testing methods. This review describes the impact of TLA in the laboratory efficiency improvement, as well as a new fully automated solution for AST by disk diffusion testing, and summarizes the evidence that implementing these methods can impact clinical outcomes.

## Introduction

The early 19^th^ century was characterized by the development of a large number of industry plants. Improving production performance – and ultimately automating the processes – was a constant motivation in industrial development, mostly for costs issues, but sometimes also for the workers’ safety. During the 20^th^ century, the electronics revolution permitted automation to alleviate humans from physically challenging, tedious, and routine handling tasks. Automation then appeared the way for companies to improve their productivity rates without increasing their employee headcounts. Reducing operating costs, improving goods availability, producing with higher reliability and increased performance are the other obvious benefits commonly expected from automation. But despite a broad consensus about those benefits, several pitfalls and a number of obstacles have to be overcome before implementing automated systems, and this holds true across various business area (Dolci et al., 2017). As the automation becomes reality, several issues concerning acceptance by the workers and their relation towards machines are surfacing. A large number of workers consider the implementation of automated systems as a direct threat to their employment (Lippi and Da Rin, 2019). Hence, the success of automation projects lies also on the way to manage staff implication as well as to identify and address their concerns. Nowadays, the automation is effectively implemented in nearly every business area, including medical laboratories (Burckhardt, 2018).

Clinical bacteriology has always been very manual and labor intensive. Unlike some other disciplines including clinical chemistry, molecular biology, immunology and hematology, total automation in clinical bacteriology is not an easy task. The level of efficiency of an automated system in clinical bacteriology relies heavily on in its potential to deal with the highly heterogeneous clinical specimens, due to various container types as well as complex analytical procedures (Bourbeau and Ledeboer, 2013; Thomson and McElvania, 2019).

The first stand-alone automated systems in clinical bacteriology were launched in the 1970s. They were designed to detect bacterial growth in blood culture specimens using broth-based cultures (the BACTEC™ 225, BACTEC™ 301, and BACTEC™ 460 were released between 1971 and 1974). The major difference between these systems and the manual operations, which were still widely used at this period, was that microbial growth was detected automatically by the BACTEC™ instrument rather than by the visual inspection of the technologist. The automatic detection of microbial growth was initially performed by adding radioactively labeled substrates to the broth. The metabolism of these substrates led to the release of radioactively-labeled carbon dioxide that is specifically detected by the instrument. When the amount of the radioactively-labeled carbon dioxide reaches a pre-defined level, the bottle is considered as suspect of microbial growth. This principle has been perpetuated in most recent blood culture instruments, with the exception that the radioactive-labeling has been replaced by fluorescence (Mitchell and Ryan, 1993; Diekema and Marchesseault, 1999).

The first total laboratory automation (TLA) system for culture-based testing (BD Kiestra™) was installed in a diagnostic laboratory in 2006. Few years after, the Copan Company commercialized another TLA system (WASPLab™) with a first installation in a routine laboratory in 2012 (Croxatto et al., 2016). The two TLA systems consist in a complex integration of robotics, digital imaging and software to pilot instruments and provide data interpretation. They are designed for accessioning and inoculating clinical specimens on a variety of culture plates, moving such media plates to automated incubators and providing high-resolution digital imaging at pre-defined time points of the culture plates. Viewing the digitized images of all plates from the same sample on a screen constitutes one of the most important advantages that automation brings to the technologists who are used to retrieve the plates from the incubators, and manipulate the agar plates to inspect them visually before executing some quick and basic biochemical tests (e.g., oxidase, catalase, Pastorex™ …). The TLA systems now enable the staff to call up the agar plates to the workbench for manual assessment whenever deemed necessary, which effectively enhances the staff’s confidence in the digitized images and minimizes handling. The workflow is also impacted by the automation. It allows extended flow processing, more compatible with a 24/7 operation, in contrast to the traditional manual approach of batch reading the plates in the morning and processing them in the afternoon. Since a large number of repetitive tasks are carried out by machines, some staff can be redirected to perform other tasks that typically require their skills and knowledge.

## Total Laboratory Automation (TLA)

### Processing of Clinical Specimens

Enhancing efficiency and specimen traceability is clearly expected from TLA. Upon receipt in the laboratory, all specimens are treated serially, without delay. Applicable culture media are selected and labeled automatically according to the specimen type, and the requested analyses. The inoculation of specimens is accurately carried out using calibrated metallic loops according to a defined streaking pattern. Inoculated media are swiftly transferred to the incubators *via* conveyors. This allows skipping the batch processing of specimens as well as the creation of manual worklists. TLA also increases the safety level of the technologists by massively reducing the handling of the specimens, especially since the inspection of culture plates is performed on screen, through digital images.

### Incubation and Imaging of Culture Media Plates

By using TLA, inoculated media are transferred without delay from the processing area to the incubators. The agar plates are incubated under optimal growth conditions, stable temperature and adequate atmosphere, because the incubators’ doors are kept closed throughout the incubation process. Microbial growth is monitored through high-resolution digital images taken at pre-defined time points. This enables a more rapid detection of microbial growth while also improving the recovery of slow-growing pathogens. Moreover, TLA uses a software that enables the digital images to be viewed under increased magnification, thus facilitating a consistent assessment of colony morphologies and the detection of mixed cultures. The digital images are interpreted by training technologists, a factor that raises an important challenge as compared to the conventional diagnostic work-up, because the bacterial colonies appear quite different on the screen. Adaptation will be necessary. TLA finally builds a library of images that can be used for training objectives, but also to support and document individual patient’s analyses during discussions with infectious disease specialists, whenever needed.

### Work-up of Culture Media Plates

**Figure 1:**
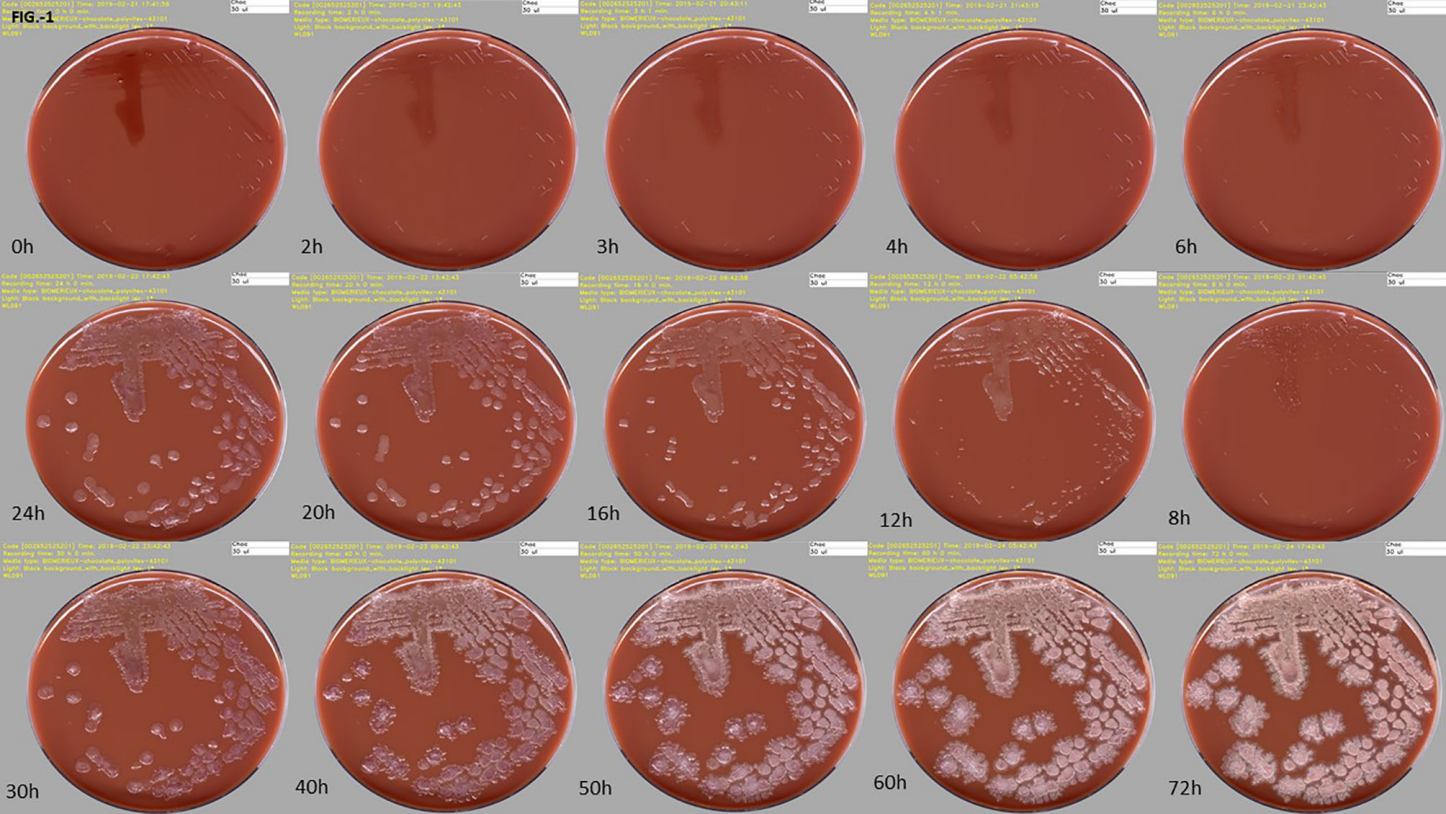
High-resolution digital images of one culture media plate taken at different incubation times on the WASPLab™. Time-series image acquisitions for a clinical specimen positive with *Bacillus* sp., at ascending time points, is depicted here, highlighting the progressive growth of bacterial colonies. Source of all images: A. Cherkaoui, Geneva University Hospitals.

**Figure 2:**
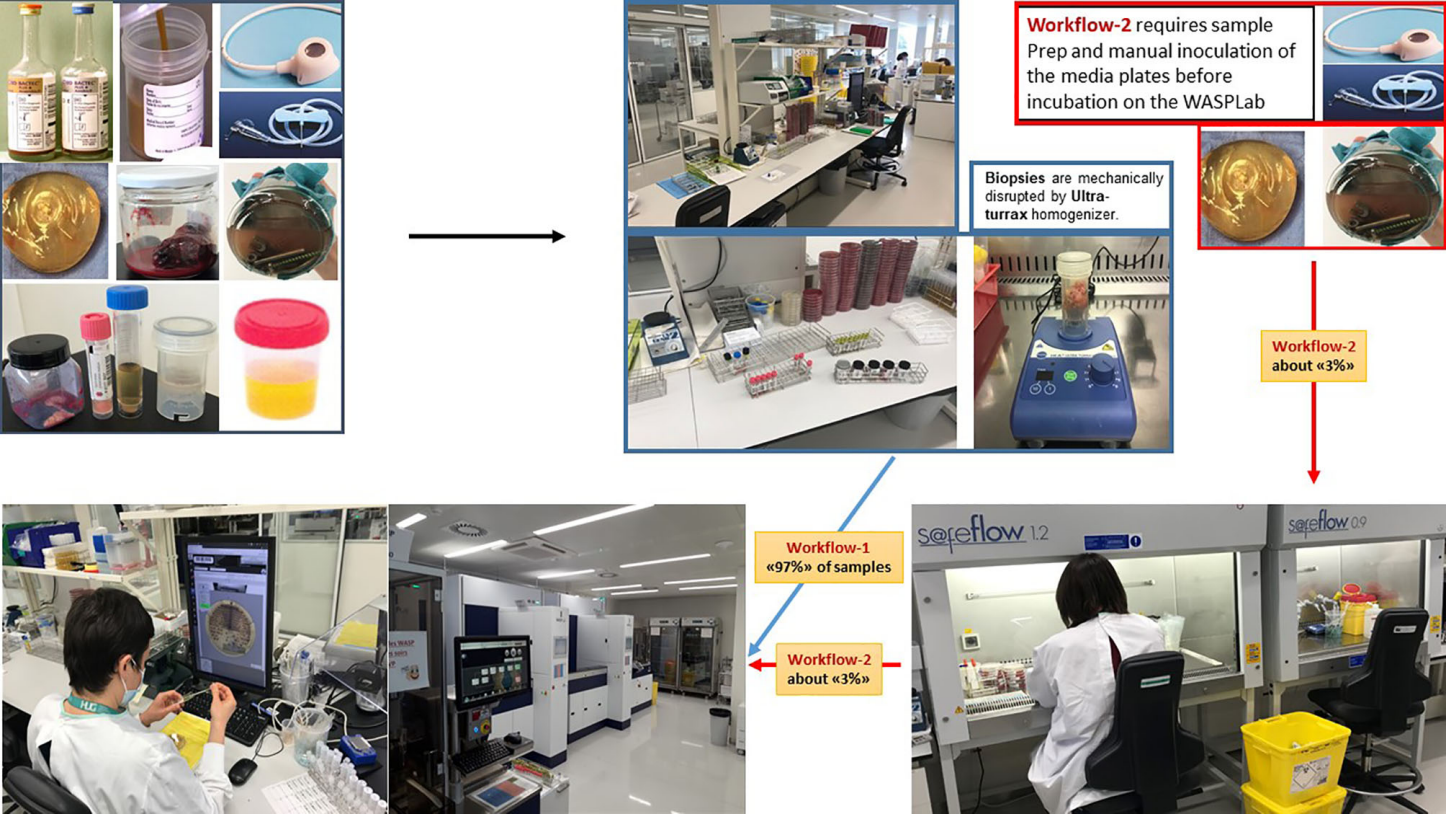
Total Lab automation workflows. A supposed advantage of TLA in microbiology is an improved efficiency by reducing the repetitive tasks with moderate added-value. Automated incubators with digital imaging drastically reduce the number of manipulations of the culture media plates. Currently, 97% of the identified “automatable” specimens are processed by TLA in our lab. The residual 3% corresponds to specimens that require manual sample preparation or manual inoculation of the media plates before incubation on the WASPLab (e.g., catheters, vascular or orthopedic prostheses, and chirurgical devices). Source of all images: A. Cherkaoui, Geneva University Hospitals.

**Figure 3:**
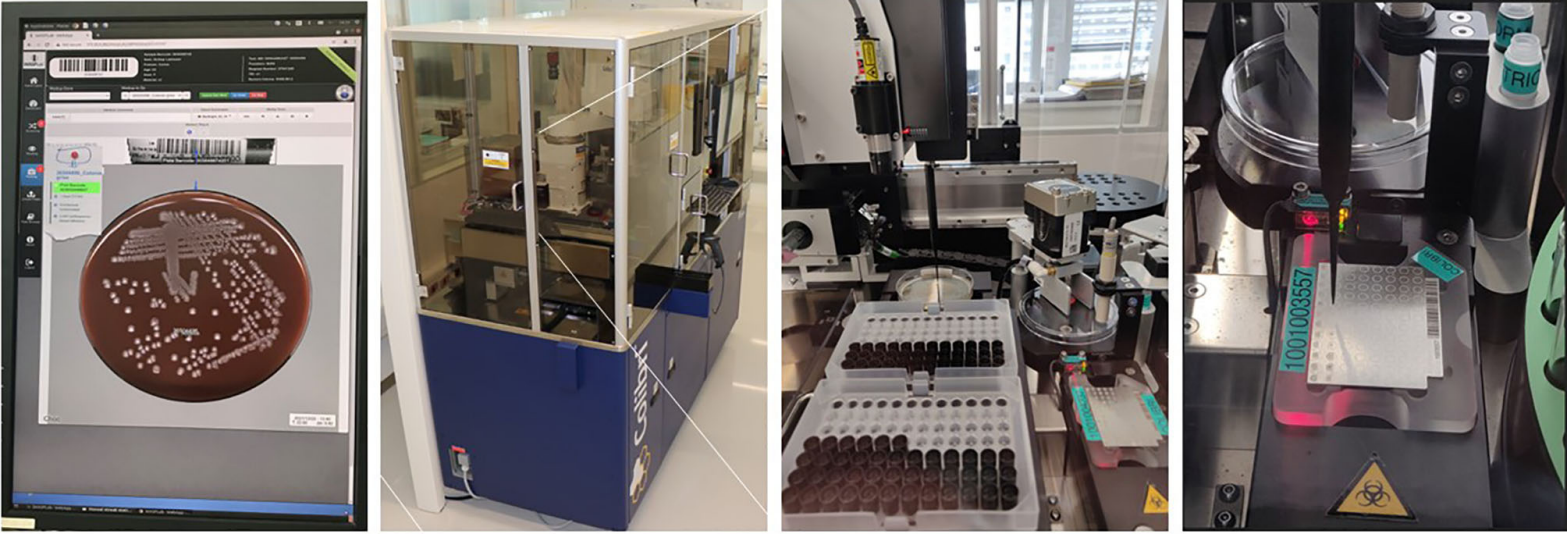
Automated System for Colony Picking and MALDI-TOF Targets Preparation (Copan Colibrı™́ ). A pipetting system permits: i) the picking of the specific colonies determined by the technologists during the reading step on the WASPLab screen; ii) the transfer of the microorganisms’ cells on the MALDI-target; and iii) the deposition of the matrix. Two protocols are available with (or without) extraction using a formic acid. Source of all images: A. Cherkaoui, Geneva University Hospitals.

To get most out of the imaging, the digital images should be performed at different time points while observing a short lead time to detect significant microbial growth as early as possible (**Figure 1**). The identification of pathogens and AST can therefore be obtained much earlier as compared to the conventional work-up, improving therefore the turnaround-time (TAT). Before the TLA era, in most clinical bacteriology laboratories, the working day starts by distributing all incubated culture plates to the different workbenches according to specimen’s type (e.g., blood cultures, urine, non-sterile site specimens, sterile site specimens, respiratory tract specimens, specimens screened for a defined microorganism carriage…) or based on patients ID or wards (ICU, ED, etc.). The specimen-based organization allows executing repetitive tasks in a more systematic manner than based on patients ID or wards and permits a better planning and follow-up of the routine activities. However, it also leads to an uneven distribution of work across workbenches, and to the difficulty to get a comprehensive diagnostic picture for an individual patient. Using TLA, each inoculated media plate is incubated for a pre-defined period, so that it becomes available for processing throughout the day and evening. This processing is most efficient and has a beneficial impact on TAT, allowing better management of the workload and of the workflow (**Figure 2**). The assessment and interpretation of culture media plates using digital images are still performed by the technologists who define which colonies of interest have to be isolated and further processed for identification and AST. The current version of TLA cannot replace these skilled activities. However, other automated systems newly introduced on the market (e.g., Copan Colibri™), can prepare the target for microbial identification (ID) by MALDI-TOF and a standardized inoculum for performing the AST (**Figure 3**). The ID and AST results must also be interpreted by skilled technologists, which requires adequate coordination in the staffing of these different activities. To help matching the workload with the level of diagnostic activity, TLA allows to track, at any moment, all specimens throughout their diagnostic pathway. TLA provides also different metrics to evaluate the processes and the team efficiencies, and additional interpretation of patient’s results including trends analysis of antimicrobial resistance.

### Implementation and Deployment

Clinical microbiology laboratories are nowadays confronted with many different challenges including the needs: i) to enhance efficiency (i.e. to deliver more cost-effective diagnostics), ii) to deliver earlier results (i.e. to shorten the TAT), iii) to comply with the increasingly demanding accreditation requirements (i.e. to provide traceability and documentation to assess the quality of the whole diagnostic process), and iv) to meet the challenges raised by the growing number of multidrug resistant organisms (i.e. to deliver rapidly more comprehensive AST, but whenever needed). Most such challenges can be significantly addressed by TLA. However, successful implementation of TLA requires : i) substantial changes in the traditional workflow, ii) strong leadership skills with smooth teamwork and cooperation of all stakeholders involved in the project, and iii) a customized support of the technical staff throughout the transition period.

## Improving Turn-Around Times (TAT) in Clinical Bacteriology

**Figures 4:**
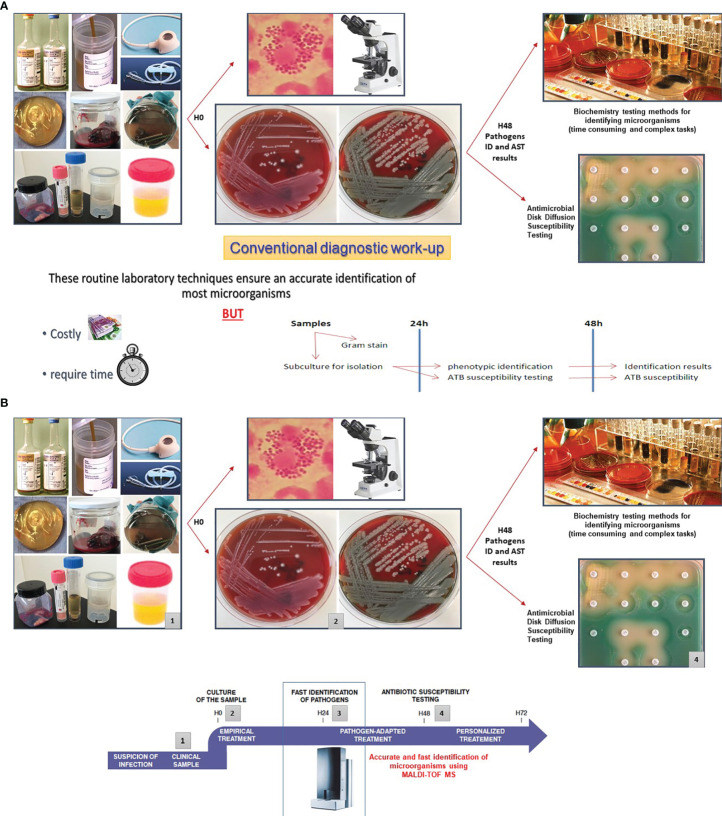
Improving Turn-Around Times (TAT) in clinical bacteriology by MALDI-TOF/MS. Several studies have highlighted that MALDI-TOF/MS is highly accurate to identify bacteria and yeasts isolated from clinical specimens. This approach is efficient, inexpensive, and rapid. The positive impact of the MALDI-TOF/MS in the enhancement of turn-around times (TAT) was assessed in various reports. **(A)** Depicts the traditional workflow using manual inoculation of the culture media plates, identification of the pathogen by traditional phenotypic methods, and manual AST by disk diffusion. **(B)** MALDI-TOF/MS reduces the TATs by an average of 1.45 days in comparison with the traditional phenotypic methods used for identification (Tan et al., 2012). Hence, this approach contributes to swiftly support treatment decisions, especially when the identification of the pathogen is unexpected (Angeletti et al., 2015; Verroken et al., 2016; Theparee et al., 2018). Source of all images: A. Cherkaoui, Geneva University Hospitals.

During the last two decades, significant resources were allocated by the industry to develop accurate and faster assays to reduce TAT in clinical bacteriology. Such timely patient management has become even more critical with the steadily increase of antimicrobial resistance. Before the TLA era, the development of matrix-assisted laser desorption/ionization time-of-flight (MALDI-TOF) mass spectrometry (MS) for the identification of bacteria, mycobacteria, yeasts, and molds has fundamentally replaced the well-established conventional diagnostic methods for identifying microorganisms, which were based essentially on biochemistry testing. In comparison with the conventional phenotype methods or molecular assays used in the identification of microorganisms, MALDI-TOF/MS has several advantages which can be summarized in three points: rapid turnaround time, low specimen volume requirements, and moderate reagent costs (Cherkaoui et al., 2010; Cherkaoui et al., 2011; Gaillot et al., 2011; Kaleta et al., 2011; Tan et al., 2012; Barberis et al., 2014; Verroken et al., 2016; Faron et al., 2017; Chen et al., 2021; Torres-Sangiao et al., 2021). Accurate and rapid identification of microorganisms using MALDI-TOF/MS helped to quickly support treatment decisions, especially when the infecting pathogen was unexpected. Thus, this technology has enabled real improvement in antimicrobial therapy, infection prevention and control measures (**Figures 4A, B**).

## Automation of Antimicrobial Susceptibility Testing (AST) by Disk Diffusion

Success criteria for TLA should be rapidly visible and related to the objectives defined before the initiation of the project. In addition to the measurable indicators exposed in the previous chapters, we considered also the implementation of a fully automated solution for the antimicrobial susceptibility testing as one of the major indicators of success for our automation project. It is even more relevant in our case because we added a second automation line with the specific purpose to automate AST by disk diffusion. Firstly, we performed an important validation study in which we investigated the agreement at the categorical level between the previous version of the automated AST by disk diffusion proposed by Copan against the SIRscan 2000 automatic, which represents a gold standard in our routine diagnostic. A large panels of resistant and susceptible strains were included in this study. The analysis revealed that the overall categorical agreement between the compared methods yielded to 99% (Cherkaoui et al., 2020). Unfortunately, the implementation of this new method, fully automated, was postponed partly owing to recurrent mechanical issues. The most important breakdown of the system was linked to the antibiotic disk dispensers and the heterogenous quality of the springs contained in the dispensers of the antibiotic disk cartridges. To overcome these issues, the manufacturer developed a new module including a carousel that can hold fifty antibiotic cartridges. This new module was developed also with the explicit goal to minimize the workflow bottlenecks on the AST line by adding a second conveyor belt for the media plates. This new fully integrated and automated system permits: to prepare an inoculum suspensions using au minimum four different colonies in order to capture different resistant patterns; to automatically inoculate the suspensions of bacterial cells over the entire surface of the specific media plates; to dispense antibiotic disks according to predefined panels; to transport the culture media to the incubators; to perform a high-quality digitalized images of the media plates at predefined time point; and finally to extract and interpret the inhibition zones diameters for all the antibiotics tested.

The basic rules to be complied when we seek to assess the accuracy of the fully automated solution for the AST by disk diffusion may be summarized in two points: i) To evaluate the ability of this new method to detect the most important resistance mechanisms, a representative number of non-duplicate clinical strains that expressed a resistant patterns to different classes of antibiotics should be included in the study; II) To determine the percentage of the major errors, mistakenly interpreted as resistant, a great number of non-duplicate susceptible clinical strains should be also included in the analysis. In our previous study we determine the agreement at the categorical level between the fully automation solution proposed by Copan for AST by disk diffusion against the Vitek2 system, which represent one of the principal AST methods used in our routine diagnostic. In this study, we included 718 strains comprising *Staphylococcus aureus*, coagulase-negative staphylococci, *Enterococcus faecalis, Enterococcus faecium*, *Pseudomonas aeruginosa*, and different species of Enterobacterales. All these strains were isolates in our laboratory from non-consecutive clinical specimens. The results of this study showed that the overall categorical agreement between the two compared methods was 99.1%. More importantly, this study revealed a great number of very major errors, mistakenly interpreted as susceptible, for *P. aeruginosa* when the AST were performed by Vitek2.

All these very major errors were linked to the hetero-resistant subpopulations within the bacteria cells (Cherkaoui et al., 2021).

## Routine AST of Anaerobes and Multidrug Resistant Gram-Negative Bacteria

**Figure 5:**
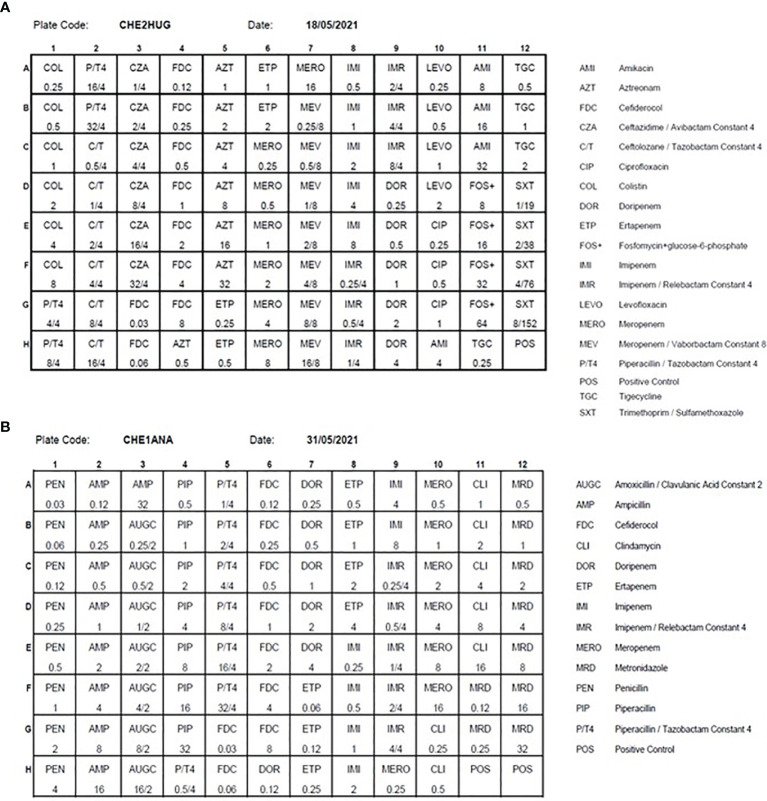
The antibiotics panels of the MDR (CHE2HUG / Panel **A**) and anaerobes (CHE1ANA / Panel **B**) Thermo Scientific™ Sensititre™ plates Source of all images: A. Cherkaoui, Geneva University Hospitals.

Over the last decade, several reports have highlighted the steadily increase in antibiotic resistance among anaerobic bacteria. The first bacteria species concerned is *Bacteroides fragilis.* In addition, antibiotic resistance is now also detected among different species that were, until recently, spared by this concern (e.g., *Clostridium difficile, Cutibacterium acnes)*. Consequently, clinicians raised concerns regarding the appropriateness of the empirical therapy (Nagy et al., 2011; Boyanova et al., 2015; Kim et al., 2016). Facing such a challenge, it was essential to perform the susceptibility testing of anaerobic bacteria routinely. As a complement to the fully automated disk diffusion, we tested on a large number of clinically relevant anaerobic strains the accuracy of the Thermo Scientific™ Sensititre™ Anaerobe MIC plate by comparing it with the ATB ANA^®^ test (BioMérieux), our current routine method. The overall categorical agreement between both methods reached 95%, allowing us to implement the Thermo Scientific™ Sensititre™ Anaerobe MIC plate (Cherkaoui et al., 2018). We then decided to design a new plate for multidrug resistant Gram-negative bacteria by integrating the latest molecules used in targeted therapy (**Figure 5A**). This plate was manufactured by Thermo Scientific*™* and validated using reference ATCC strains. This plate is now implemented as our second line testing, whenever AST by disk diffusion has detected defined resistance patterns. This strategy enabled us to effectively monitor antibiotic resistance in our institution at a reasonable cost, by systematically targeting suspect strains, as reported by the automated AST testing. Finally, we also designed a new plate for the routine susceptibility testing of anaerobes, which the antibiotics panel fits better with the treatment guidelines. We included in this panel some new antibiotics, to anticipate the emergence of new resistance mechanisms within this group of bacteria (**Figure 5B**).

The Sensititre^®^ susceptibility system is a micro broth method using dried plate format. Qualitative (susceptible or resistant) and quantitative Minimum Inhibitory Concentration (MIC) results are provided. Dried plates are dosed with serially diluted antimicrobial agents selected for the micro-organisms tested. Strain suspensions are prepared manually and the plates are automatically inoculated using Sensititre AutoInoculator^®^/AIM^®^. After incubation, results are read using the Sensititre**^®^** manual viewer. Bacterial growth occurs as turbidity or as a deposit of cells at the bottom of a well. The MIC is defined as the lowest concentration of drug that inhibits visible bacterial growth.

Over the last few decades, multidrug-resistant Gram-negative bacterial infections have become one of the major areas of concern in medicine and global health. To optimize the selection of a successful treatment and to deal with the increasing number of carbapenemase-producing Gram negative bacteria, the additional use of Minimum Inhibitory Concentration (MIC) results for last resource drugs has become essential. This allows targeted AST determinations on MDR strains and, when coupled to therapeutic drug monitoring (TDM), permits to select the most appropriate treatments. As agar disk diffusion does not provide MICs, the Sensititre constitutes an interesting complement to the fully automated AST by disk diffusion when deemed necessary, as in the case of MDR strains or for anaerobic bacteria.

## Discussion

All clinical microbiology laboratories around the world express the unmet need for improved quality, productivity, reduced turnaround-time (TAT), rationalization of laboratory technologist labor force, and faster diagnostics to reduce the misuse of antibiotics. Responding to these growing needs will require highly effective strategies. In that sense, the implementation of TLA represents an appealing option, given the fact that the bulk of the workload in culture-based analysis relies in specimen handling, media plate inoculation, incubation, culture reading, pathogen identification and antimicrobial susceptibility testing (ID/AST), all of which remaining highly manual processes.

As discussed in the previous chapters, TLA enables rapid results and significantly reduces TAT. In 2020, about 30’000 urine specimens were processed in our lab. Using TLA, the median TAT for negative reports decreased by almost half from 52.1 h (2017) to 28.3 h (2019) (p < 0.001), potentially allowing to stop unnecessary antimicrobial treatments (Cherkaoui et al., 2020). It is important to note that this reduction of TAT was not associated with a loss of analytical sensitivity. Rather, automated processing of urine specimens and optimized incubation periods contributed to higher detection rates for fastidious uropathogens such as *Alloscardovia* spp. and *Aerococcus* spp (Klein et al., 2018; Lainhart and Burnham, 2018; Cherkaoui et al., 2019). It is noteworthy to mention that the benefits of TLA for patient care (including drug de-escalation or antimicrobial therapy adjustments) should be further improved, providing trained technologists are available to analyze the on-screen digital images and to report positive cultures results in real time, which would require 24/7 staffing. This represents a real challenge for many laboratories, in terms of costs but also of attracting trained technologists. This is why artificial intelligence (AI), like automated colony recognition, is making its way towards routine microbiology, as it can be coupled to TLA. These applications compare media plate images at time zero and at defined incubation time points in order to identify potential bacterial growth, but also to differentiate bacterial morphologies and enumerate corresponding colonies (Van et al., 2019; Foschi et al., 2021). After careful validation studies, negative cultures may be automatically processed and the results automatically released without human intervention, which further increases the laboratory efficiency and improves the TAT (Faron et al., 2019; Faron et al., 2020; Ford and McElvania, 2020; Dauwalder et al., 2021).

To face the continual increase of antimicrobial resistance and its dramatic consequences, infection control has become a core activity of clinical microbiology labs. The amount of screening specimens for carbapenemase-producing Enterobacteriaceae (CPE), extended-spectrum β-lactamases (ESBLs), methicillin-resistant *Staphylococcus aureus* (MRSA), and vancomycin-resistant *Enterococcus* VRE carriage sent into the microbiology labs for analysis has increased thoroughly over the last decade.

In our laboratory, we process about 35’000 screening specimens per year. Using TLA, the median TAT for negative reports decreased by almost half for MRSA screening from 50.7 to 26.3 h (p < 0.001). The difference in median TAT for negative cultures was less pronounced for screening of ESBLs (50.2 vs. 43.0 h) (p < 0.001) and VRE (50.6 vs. 45.7 h) (p < 0.001), but remained significant (Cherkaoui et al., 2020). Several recent reports have also highlighted the positive impact of the TLA on the TAT (Cheng et al., 2020; Zhang et al., 2021; Kim et al., 2022).

Upcoming improvements in TLA will enable positive cultures to be automatically released for MRSA or VRE when chromogenic media are used and analyzed by artificial intelligence algorithms. These algorithms permit recognition of characteristically colored colonies, with performances that can be measured and whose cut-offs can be customized by the labs (Faron et al., 2016a; Faron et al., 2016b). Shortening the TAT could prevent the spread of multi-resistant bacteria and reduce nosocomial transmission, by alleviating unnecessary infection control measures or by prompting specific searches whenever appropriate. Yet, despite these major improvements, the real impact of TLA on medical decisions stumbles upon the reactiveness of the medical teams when the results are delivered by the laboratory information system or when pushed to the physicians’ cell phones.

In the last few years, many countries have experienced a lack of trained microbiological technologists. This problem is further emphasized in the USA. The direct consequences of this shortage in skilled technologists leads to understaffed diagnostic laboratories, which is reflected in delayed analysis results and increased risks for patients (Culbreath et al., 2021; Zimmermann, 2021). Likewise, laboratories have progressively lost their ability to develop customized diagnostic procedures when facing complex cases, not to mention their capability and delay to detect them. In response to this increasing problem, one approach is to optimize the productivity by increasing the number of clinical specimens handled per FTE without compromising the quality nor overloading the staff. TLA has made this possible. Moreover, TLA is devoted to accommodate the constantly increasing demand for clinical bacteriology analyses and to empower the specialized skills of the laboratory technologists by allowing them (i) to dedicate more of their time to the analysis of complex specimens, (ii) to execute new tasks including system troubleshooting (both the instrument and its software), and (iii) to establish, and validate new protocols.

The economic benefits of the implementation of TLA can be measured throughout the increased productivity accompanied by a reduction of the cost per specimen as reported by Culbreath et al. (2021). In this report, the authors determined the economic benefits of the implementation of TLA in four different-sized clinical microbiology labs in North America. They observed that the productivity increased by up to 90%, while the cost per specimen was reduced by up to 47% by using TLA. These measurable improvements led to annual laboratory savings of up to $1.2 million, depending on the size of the lab operations. The integration of the upcoming artificial intelligence tools will ensure further improvement in the efficiency and the quality of the results (Culbreath et al., 2021).

For several years, before the implementation of more stringent laboratory regulation, the practice of using the positive blood culture broth as the inoculum for AST by disk diffusion and some semi-automated AST methods was widely used to speed up the AST results from positive blood cultures, with an acceptable accuracy. The important factors in favor of this method were the following: i) when growth is detected in a positive blood culture, the concentration of bacteria approximates a 0.5 McFarland, and ii) the overwhelming majority of positive blood cultures is mono-microbial (Banerjee and Humphries, 2021). Nowadays this practice becomes less frequently carried out in modern microbiology laboratories, largely due to the actual laboratory regulations and the arrival of new EUCAST guidelines for rapid AST directly from positive blood culture bottles. This method enables the reading of the inhibition zones of certain drugs as early as 4, 6 and/or 8 hours. The performance of this method has been widely assessed in different centers. Thus, 70% and 80% of the inhibition zones diameters can be accurately interpreted at 4h and 6h, respectively (Akerlund et al., 2020; Jonasson et al., 2020). Overall, the EUCAST rapid AST directly from positive blood culture method remains time consuming and its interpretation is more error-prone. However, the advent of the fully automated antimicrobial disk diffusion susceptibility testing on TLA should facilitate the implementation of this method by accommodating this continuously increasing activity with a minimal workload and a high traceability.

## Conclusions

TLA has now shown able to accommodate the high diversity and complexity of the procedures required for culture-based testing in clinical microbiology laboratories. Its positive impact on various metrics (clinical challenges, productivity, traceability, quality management, and TAT) have also been thoroughly assessed and published. These advantages have most recently been reinforced by the availability of full automation of AST by disk diffusion.

The implementation of artificial intelligence (AI) to rapidly identify bacterial growth (detection), but also to differentiate bacterial morphologies (segmentation) and enumerate the corresponding colonies (counting) will further enhance the workflow and ensure reproducible and predictable performances. Careful validation studies are now warranted to enable negative cultures to be automatically processed using AI and its results automatically released without human intervention. Finally, TLA systems (using the Colibri coupled to the Radian) will facilitate the implementation of EUCAST rapid AST which can bring several advantages regarding patient outcomes.

## Author Contributions

AC designed the review and wrote the manuscript. JS revised the manuscript. All authors contributed to the article and approved the submitted version.

## Funding

This study was supported by internal funding.

## Conflict of Interest

The authors declare that the research was conducted in the absence of any commercial or financial relationships that could be construed as a potential conflict of interest.

## Publisher’s Note

All claims expressed in this article are solely those of the authors and do not necessarily represent those of their affiliated organizations, or those of the publisher, the editors and the reviewers. Any product that may be evaluated in this article, or claim that may be made by its manufacturer, is not guaranteed or endorsed by the publisher.
